# Dose–response relationship between Chinese visceral adiposity index and type 2 diabetes mellitus among middle-aged and elderly Chinese

**DOI:** 10.3389/fendo.2022.959860

**Published:** 2022-10-05

**Authors:** Liang Pan, Qianqian Xu, Jianmin Liu, Yang Gao, Jun Li, Hongye Peng, Linli Chen, Miyuan Wang, Gang Mai, Shuo Yang

**Affiliations:** ^1^ Phase 1 Clinical Trial Center, Deyang People’s Hospital, Deyang, China; ^2^ Department of Medical Imaging Center, Zhongnan Hospital of Wuhan University, Wuhan, China; ^3^ Department of Otolaryngology and Head and Neck Surgery, Deyang People’s Hospital, Deyang, China; ^4^ Department of Pediatrics , Deyang People’s Hospital, Deyang, China; ^5^ Department of Nephrology , Deyang People’s Hospital, Deyang, China; ^6^ Graduate School, Beijing University of Chinese Medicine, Beijing, China; ^7^ School of Public Health, Huazhong University of Science and Technology, Wuhan, China; ^8^ Central Laboratory, HuangGang Hospital of Traditional Chinese Medicine (TCM), Huanggang, China

**Keywords:** chinese visceral adiposity index, type 2 diabetes mellitus, dose–response relationship, restricted cubic spline regression, prospective cohort study

## Abstract

**Introduction:**

China has the largest population of diabetic patients (about 116 million) in the world. As a novel model of the fat index for Chinese people, the Chinese visceral adiposity index (CVAI) was considered a reliable indicator to assess the dysfunction of visceral fat. This study aimed to explore the dose–response relationship between CVAI and type 2 diabetes mellitus (T2DM) in the Chinese population, considering CVAI as a continuous/categorical variable.

**Method:**

Baseline and follow-up data were collected from waves 2011 and 2015, respectively, of the China Health and Retirement Longitudinal Study (CHARLS). Multivariate logistic regression models were used to explore the relationship between CVAI and T2DM. We built three models to adjust the possible effect of 10 factors (age, gender, education level, location, marital status, smoking status, drinking status, sleep time, systolic blood pressure (SBP), and diastolic blood pressure (DBP)) on the outcome. The restricted cubic splines were used to examine possible non-linear associations and visualize the dose–response relationship between CVAI and T2DM.

**Results:**

A total of 5,014 participants were included, with 602 (12.00%) T2DM patients. The last CVAI quartile group (Q4) presented the highest risk of T2DM (OR, 2.17, 95% CI, 1.67–2.83), after adjusting for all covariates. There was a non-linear (U-shaped) relationship between the CVAI and the risk of T2DM (*p* for non-linear <0.001) in the restricted cubic spline regression model. CVAI was a risk factor of T2DM when it exceeded 92.49; every interquartile range (IQR) increment in the CVAI was associated with a 57% higher risk of developing T2DM (OR = 1.57, 95% CI = 1.36–1.83) after adjusting for potential confounders. The area under the receiver operating characteristic curve (AUC) (95% confidence interval) for CVAI was 0.623, and the optimal cutoff point was 111.2. There was a significant interaction between CVAI and gender by stratified analysis.

**Conclusion:**

CVAI was closely associated with the risk of T2DM and might possibly be a potential marker in predicting T2DM development. The outcome suggested that it might be better to maintain CVAI within an appropriate range.

## 1 Background

Type 2 diabetes mellitus (T2DM) is a group of metabolic diseases characterized by hyperglycemia and is closely associated with a variety of factors, such as genes, obesity, and lack of exercise. The incidence of T2DM is rising rapidly due to living standards improvement, changes in diet, sedentary lifestyle, and work-related stress. According to the International Diabetes Federation (IDF), the population with diabetes mellitus worldwide is about 537 million presently and is expected to reach 700 million by 2045 (1). Diabetes mellitus may contribute to retinopathy (2), foot ulcer and necrosis (3), cardiovascular disease (4), myocardial infarction, and stroke, which may seriously endanger human health and impose a heavy burden on society. Based on the epidemiological and economic data from 184 countries (5), the global cost associated with diabetes mellitus is about 1.31 trillion USD in 2015 (95% CI: 1.28–1.36). On average, such cost as a percentage of GDP in middle-income countries is higher than that in high-income countries. According to IDF, China has the largest population of diabetic patients (about 116 million) and spends over 109 billion USD on treatment in 2021, which is the largest expenditure in the world outside the United States (6). A large population of diabetic patients and associated substantial costs may impose a heavy burden on society in China. Over 90% of those diabetic patients are T2DM patients. Therefore, it is crucial to lower the incidence of T2DM and improve overall health by identifying high-risk populations and implementing early intervention and treatment.

According to the WHO, there are 650 million obese adults worldwide in 2021 (7). It is known that obesity, especially abdominal obesity, predisposes the development of insulin resistance and T2DM (8). However, commonly used indicators, such as body mass index (BMI), waist circumference (WC), and waist-to-height ratio (WHtR), are unable to differentiate visceral and subcutaneous fat. Visceral adiposity index (VAI), a novel fat fraction calculated with WC, BMI, and serum lipid, is closely associated with multiple chronic metabolic diseases, such as coronary heart disease (9), hypertension (10), and T2DM (11). Compared with conventional indices, VAI has a higher predictive value for obesity. It is known that the degree and susceptibility of obesity vary in different ethnicities and regions (12). Chinese people are more likely to have abdominal obesity compared with Western populations, which makes VAI an inadequate predictor for Chinese people. Based on VAI, Xia et al. proposed the Chinese visceral adiposity index (CVAI) as a novel model of the fat index for Chinese people, integrating parameters of age, BMI, WC, triglyceride (TG), and high-density lipoprotein cholesterol (HDL-C). CVAI is considered a reliable index to assess the dysfunction of visceral fat (13).

CVAI has been reported with predictive value in previous studies. Based on research in rural China, Han et al. (14) suggested that CVAI is positively associated with the risk of T2DM. Shang et al. (15) explored the relationship between CVAI and the risk of newly emerging T2DM in Japanese adults. However, those studies cannot reflect the characteristics of the Chinese population due to their geographical limitations. Based on the China Health and Retirement Longitudinal Study (CHARLS) database, this prospective cohort study aims to explore the value of CVAI in predicting the risk of developing T2DM in the Chinese population, considering CVAI as a continuous/categorical variable. Moreover, stratified analysis has been performed with age, gender, marital status, and education level.

## 2 Method

### 2.1 Study population

In this prospective cohort study, we analyzed baseline data in wave 2011 and follow-up data for T2DM in wave 2015 from CHARLS (http://charls.pku.edu.cn/), a freely accessible database of a nationally representative sample.

As a nationwide interdisciplinary survey, CHARLS investigated participants aged 45 and above from 450 villages/communities in 28 provinces (autonomous regions and municipalities) and collected comprehensive data, such as basic information, family structure, physical condition, health care and insurance, work and pension, income and expenditure, housing, and laboratory examination. With a total of 21,097 participants in 12,241 households in 2015, CHARLS was a reliable database for research on the health status and possible contributing factors for the middle-aged and elderly population.

Venous blood samples were collected from participants after overnight fasting for over 12 h. A complete blood count was tested promptly on site. Whole blood specimens were stored at 4°C. All remaining samples were delivered to the central laboratory for further analysis. Enzymatic colorimetric analysis was performed to test glucose, total cholesterol (TC), TG, low-density lipoprotein cholesterol (LDL-C), and HDL-C levels. Boronate-affinity high-performance liquid chromatography method was used to test glycated hemoglobin (HbA1c) levels.

The program CHARLS was reviewed and approved by Ethical Review Committee at Peking University in 2008 (IRB00001052-11, 015). Our study followed all applicable specifications and guidelines of CHARLS. Informed consent forms were signed voluntarily by all participants before the investigation.

This study implemented the following inclusion criteria according to the research purpose: age ≥ 45 years; complete sociodemographic data including gender, education level, marital status, and location; and complete data of fasting blood glucose. Participants with lipid-lowering drug-taking history were excluded (n = 253). A total of 5,014 eligible participants were included finally.

### 2.2 Measurement

#### 2.2.1 Assessment of visceral adiposity index

VAI, an empirical-mathematical model integrating anthropometric (age, BMI, and WC) and functional (TG and HDL) parameters, indicated the fat distribution and function. The VAI score for the Chinese population was calculated with the following specific formulas:

Chinese man: CVAI = −267.93 + 0.68 × age + 0.03 × BMI + 4.00 × WC + 22.00 × log10 (TG) − 16.32 × HDL-C

Chinese woman: CVAI = −187.32 + 1.71 × age + 4.23 × BMI + 1.12 × WC + 39.76 × log10 (TG) − 11.66 × HDL-C.

#### 2.2.2 Assessment of type 2 diabetes mellitus

According to the standards published by American Diabetes Association in 2005, this study defined T2DM as a fasting blood glucose level ≥126 mg/dl (7 mmol/L), and/or a random blood glucose level ≥200 mg/dl (11.1 mmol/L), and/or an HbA1c level ≥6.5%, and/or self-reported diagnosis with yes (“Have you ever been diagnosed with diabetes or hyperglycemia?”)

#### 2.2.3 Assessment of covariates

The analyses were adjusted for sociodemographic characteristics, health-related behaviors, and anthropometric measurements. We analyzed age, gender, education level (“primary school or below”, “high school”, and “college or above”), location (“city/town” and “village”), and marital status (“married” and “never-married/separated/widowed”) as demographic variables. We analyzed smoking status (“non-smoker”, “ex-smoker”, and “current smoker”), drinking status (“never”, “less than once a month”, and “more than once a month”), and sleep time as variables of health-related behavior. Those data were obtained from self-reported questionnaires and with the help of trained interviewers. Anthropometric measurements included systolic blood pressure (SBP) and diastolic blood pressure (DBP), which were the means of the three-time measurements using Omron HEM-7200 sphygmomanometer.

### 2.3 Statistical analysis

Continuous variables with normal distribution were expressed by means and standard deviation (SD), while those with abnormal distribution were expressed by median (interquartile range (IQR)). Categorical variables were expressed by percentages. One-way ANOVA, Kruskal–Wallis H test, or chi-square tests were employed to compare the baseline characteristics and incidence of T2DM after grouping by CVAI quartiles (Q1, Q2, Q3, and Q4). Three logistic models were employed to estimate the odds ratio (OR) with a 95% confidence interval (CI) for T2DM using CVAI as continuous variables (per IQR increment) or categorical (quartiles) variables. All three models were to explore the relationship between CVAI and T2DM, including the unadjusted crude model (Model 1); the adjusted model with age, gender, education level, location, and marital status (Model 2); and a further adjusted model with smoking status, drinking status, sleep time, SBP, and DBP (Model 3). Those results were presented with ORs and 95% CIs. The area under the receiver operating characteristic (ROC) curve (AUC) was performed to test the predictive power of CVAI for the risk of T2DM, and the “addfor” algorithm was used to determine the optimal cutoff points. Interaction analysis was performed to identify the effect modifications of sociodemographic characteristics, health-related behaviors, and anthropometric measurements in the relationship between CVAI and T2DM, using a product term in the main analysis [CVAI × (interaction term)]. Moreover, the restricted cubic splines were used to examine possible non-linear associations and visualize the dose–response association of CVAI with T2DM.

All statistical analyses were completed with R 4.1. Restricted cubic splines were completed with the “rms” package; optimal cutoff points were tested with the “CatPredi” package. A two-tailed *p* < 0.05 denoted statistical significance.

## 3. Result

### 3.1 Baseline characteristics

Characteristics of the study population are presented in **Table 1**. A total of 5,014 participants were included (median age = 58, men = 2,321 (46.29%), and women = 2,693 (53.71%)), of whom 602 (12.00%) developed T2DM. The baseline median (IQR) CVAI in all participants was 91.32 (62.17, 122.07). The characteristics of participants with T2DM were obviously different from the characteristics of those without; specifically, the former were more likely to be older and non-married, with higher SBP, DBP, BMI, WC, glucose, TG, and CVAI and lower HDL-C.

**Table 1:** Characteristics of the participants at baseline (N = 5,014).

	Total (n = 5,014)	Non-T2DM (n = 4,412)	T2DM (n = 602)	*p*
Age	58 (52, 65)	58 (51, 64)	60 (53, 67)	<0.01
Sex				0.81
Female	2,693 (53.71)	2,373 (53.79)	320 (53.16)	
Male	2,321 (46.29)	2,039 (46.21)	282 (46.84)	
Marital				0.03
Non-married	569 (11.35)	484 (10.97)	85 (14.12)	
Married	4,445 (88.65)	3,928 (89.03)	517 (85.88)	
Education				0.11
Primary school or below	3,569 (71.18)	3,122 (70.76)	447 (74.25)	
High school	1,013 (20.2)	898 (20.35)	115 (19.1)	
College or above	432 (8.62)	392 (8.88)	40 (6.64)	
Location				0.97
City/town	4,725 (94.24)	4,157 (94.22)	568 (94.35)	
Village	289 (5.76)	255 (5.78)	34 (5.65)	
Smoking				0.54
Non-smoker	3,063 (61.09)	2,707 (61.36)	356 (59.14)	
Current smoker	397 (7.92)	349 (7.91)	48 (7.97)	
Ex-smoker	1,554 (30.99)	1,356 (30.73)	198 (32.89)	
Drinking				0.13
Drink but less than once a month	400 (7.98)	363 (8.23)	37 (6.15)	
Drink more than once a month	1,277 (25.47)	1,130 (25.61)	147 (24.42)	
None of these	3,337 (66.55)	2,919 (66.16)	418 (69.44)	
Sleep time	6 (5, 8)	6.5 (5, 8)	6 (5, 8)	0.88
SBP	125.67 (113.33, 140.33)	124.83 (113, 139.33)	130.5 (119.33, 145)	<0.01
DBP	74.33 (67, 82.67)	74 (66.67, 82.33)	76.67 (69.67, 85.67)	<0.01
BMI (kg/m^2^)	22.93 (20.78, 25.44)	22.8 (20.7, 25.25)	23.94 (21.49, 26.82)	<0.01
WC (cm)	84 (77.1, 91)	83.2 (77, 90)	87.4 (79.2, 94.2)	<0.01
Glucose (mg/dl)	100.26 (93.24, 107.28)	99.72 (93.06, 106.56)	104.58 (96.48, 111.73)	<0.01
TG (mg/dl)	100.89 (72.57, 143.37)	100 (71.68, 141.6)	110.62 (81.42, 158.19)	<0.01
HDL-C (mg/dl)	50.64 (41.75, 60.7)	50.64 (42.14, 61.08)	47.55 (39.05, 59.05)	<0.01
CVAI	91.32 (62.17, 122.07)	89.49 (60.72, 119.13)	109.89 (73.86, 143.04)	<0.01
Quartiles of CVAI				<0.01
Q1	1,254 (25.01)	1,138 (25.79)	116 (19.27)	
Q2	1,253 (24.99)	1,156 (26.2)	97 (16.11)	
Q3	1,253 (24.99)	1,102 (24.98)	151 (25.08)	
Q4	1,254 (25.01)	1,016 (23.03)	238 (39.53)	

T2DM, type 2 diabetes mellitus; SBP, systolic blood pressure; DBP, diastolic blood pressure; BMI, body mass index; WC, waist circumference; TG, triglyceride; HDL-C, high-density lipoprotein cholesterol; CVAI, Chinese visceral adiposity index.

### 3.2 Dose–response relationship between Chinese visceral adiposity index and type 2 diabetes mellitus

**Table 2** showed the association of the CVAI with the risk of T2DM, as well as the quartiles of the CVAI. The risk of T2DM showed a gradual increase with the quartiles of the CVAI (*p* for trend <0.001). Compared with the first CVAI group (Q1), the last CVAI quartile group (Q4) presented the highest risk of developing T2DM (OR, 2.17, 95% CI, 1.67–2.83), after adjusting for age, gender, education level, location and marital status, smoking status, drinking status, sleep time, SBP, and DBP. Analyzed as a continuous variable, CVAI was consistently and significantly associated with T2DM (OR, 1.59, 95% CI, 1.41–1.81). The AUC (95% confidence interval) for CVAI was 0.623, and the optimal cutoff point was 111.2.

**Table 2:** Association of CVAI with the risk of T2DM in the CHARLS.

	Model 1	*p*	Model 2	*p*	Model 3	*p*
CVAI per IQR	1.66 [1.47, 1.86]	<0.001	1.67 [1.48, 1.89]	<0.001	1.59 [1.41, 1.81]	<0.001
Quartiles of CVAI						
Q1	Ref		Ref		Ref	
Q2	0.82 [0.62, 1.09]	0.176	0.87 [0.65, 1.15]	0.326	0.85 [0.64, 1.14]	0.276
Q3	1.34 [1.04, 1.74]	0.023	1.44 [1.10, 1.88]	0.008	1.37 [1.05, 1.81]	0.023
Q4	2.30 [1.82, 2.92]	<0.001	2.38 [185, 3.07]	<0.001	2.17 [1.67, 2.83]	<0.001
* p* for trend	<0.001		<0.001		<0.001	

Model 1 was crude model. Model 2 was adjusted for age, gender, education level, location, and marital status. Model 3 was adjusted for age, gender, education level, location and marital status, smoking status, drinking status, sleep time, SBP, and DBP.

CVAI, Chinese visceral adiposity index; IQR, interquartile range; T2DM, type 2 diabetes mellitus; CHARLS, China Health and Retirement Longitudinal Study; SBP, systolic blood pressure; DBP, diastolic blood pressure.

**Figure 1:**
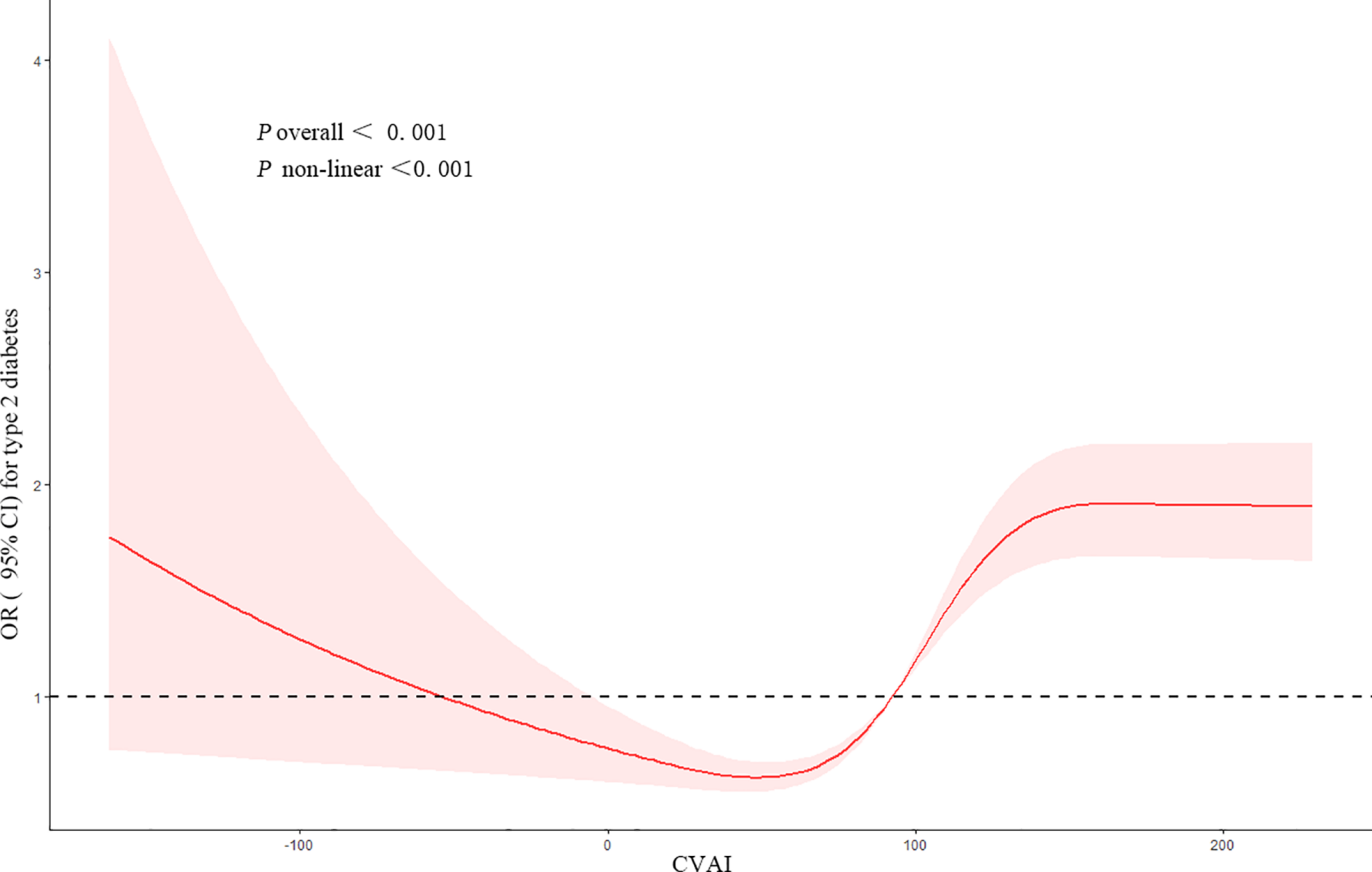
Restricted cubic spline of the association between CVAI and the risk of T2DM. The model was adjusted for age, gender, education level, location and marital status, smoking status, drinking status, sleep time, SBP, and DBP. The plot shows a non-linear (U-shaped) relationship between CVAI and the risk of T2DM. CVAI, Chinese visceral adiposity index; T2DM, type 2 diabetes mellitus; SBP, systolic blood pressure; DBP, diastolic blood pressure.

**Figure 1** showed the dose–response relationship between CVAI and the risk of T2DM. Restricted cubic spline regression showed a non-linear (U-shaped) relationship between CVAI and risk of T2DM (*p* for non-linear <0.001). We found that when CVAI ≥ 92.49, the cumulative risk of developing T2DM increased gradually, so we used piecewise logistic regression to calculate the risk of T2DM (**Table 3**). Results showed that every IQR increment in CVAI was associated with a 57% higher risk of developing T2DM (OR, 1.57, 95% CI, 1.36–1.83) after adjusting for potential confounders.

**Table 3:** Associations of CVAI with the risk of T2DM using piecewise logistic regression.

	Model 1	*P*	Model 2	*p*	Model 3	*p*
CVAI per IQR						
≥92.49	1.64 [1.42, 1.89]	<0.001	1.61 [1.39, 1.86]	<0.001	1.57 [1.36, 1.83]	<0.001

Model 1 was crude model. Model 2 was adjusted for age, gender, education level, location, and marital status. Model 3 was adjusted for age, gender, education level, location and marital status, smoking status, drinking status, sleep time, SBP, and DBP.

CVAI, Chinese visceral adiposity index; IQR, interquartile range; T2DM, type 2 diabetes mellitus; SBP, systolic blood pressure; DBP, diastolic blood pressure.

### 3.3 Stratified analysis

**Figure 2:**
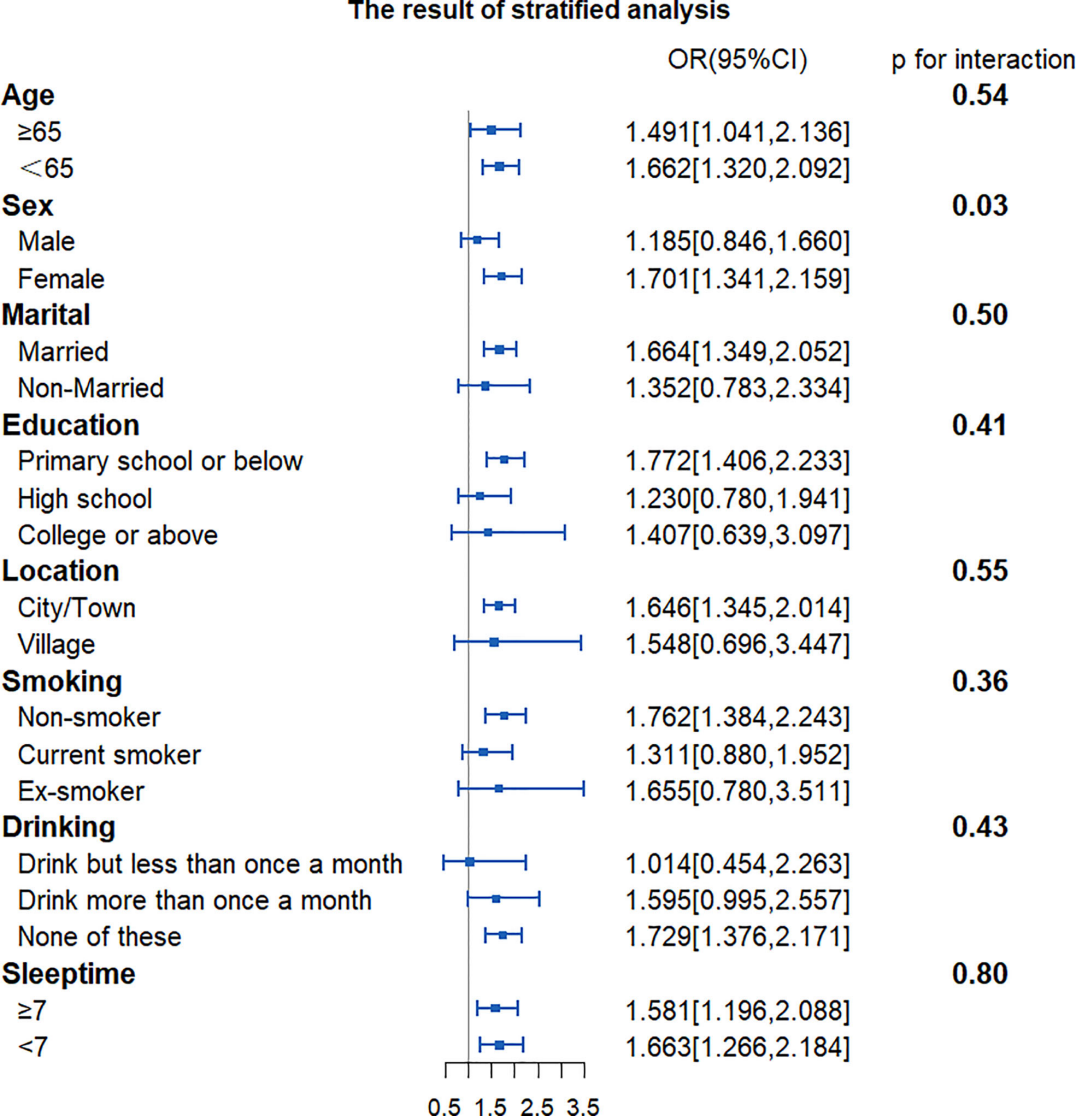
Forest plot of stratified analysis of the association of CVAI with the risk of T2DM. The plot shows that there were significant interactions between CVAI and sex. OR, odds ratio; CI, confidence intervals; CVAI, Chinese visceral adiposity index; T2DM, type 2 diabetes mellitus.

To find out whether CVAI would exert different influence on the risk of developing T2DM among different subgroups, we grouped participants by characteristics. Results showed that the influence of CVAI on the risk of T2DM was consistent across subgroups. There was a significant interaction between CVAI and gender (*p* for interaction <0.001). Each IQR increment in CVAI was associated with a 98.7% higher risk of developing T2DM (OR, 1.987, 95% CI, 1.663–2.375) for women (**Figure 2**).

## 4 Discussion

According to the baseline data of CHARLS, we found that participants with T2DM were more likely to be older and non-married, with higher SBP, DBP, BMI, WC, glucose, TG, and CVAI and lower HDL-C than those without. Based on the follow-up data, we found that CVAI was positively associated with the risk of T2DM. After adjusting for confounders, each IQR increment in CVAI was associated with a 59% higher risk of developing T2DM (OR, 1.59, 95% CI, 1.41–1.81). Based on the dose–response relationship analysis, we found a non-linear (U-shaped) relationship between CVAI and the risk of T2DM.

Obesity, especially abdominal obesity, is an important risk factor for T2DM. Our study used CVAI to predict the risk of T2DM in the Chinese population and suggested that they were positively associated, which was consistent with previous studies (16, 17). Han et al. (14) suggested that the risk of developing T2DM increased with each SD increment of CVAI, VAI, WHtR, WC, and BMI. Of all those five indices, CVAI presented the best performance in predicting T2DM, with the largest AUC. Wan et al. (18) explored the association of CVAI with T2DM and its complications (such as cardiovascular diseases, diabetic nephropathy, and fundus lesions) and suggested that CVAI was closely associated with the incidence of cardiovascular diseases and diabetic nephropathy. Different from the mentioned studies using CVAI only as a categorical variable, this study also used CVAI as a continuous variable to explore its dose–response relationship with the risk of T2DM. We also performed a stratified analysis.

When using CVAI as a continuous variable, we found that it was non-linearly (U-shaped) associated with the risk of T2DM. An appropriate CVAI level might lower the risk of T2DM, while a high CVAI level might increase the risk of T2DM. However, these results were not entirely unexpected. Wang et al. (19) suggested an inverted U-shaped relationship between VAI and lung function. For men, the predicted FEV1% and FVC% increased as VAI increased when VAI ≤ 14, while they decreased as VAI increased when VAI ≥ 15. Triglyceride-glucose index (TyG) is an important indicator of insulin resistance. Xuan et al. (20) suggested that TyG had a U-shaped association with incident T2DM. As a component for calculating CVAI, TG was also found to have a U-shaped association with the risk of T2DM (21). Interestingly, a 5.5-year prospective cohort study with a sample of 149,345 cases suggested that long-term overweight (BMI 25 to <30 kg/m^2^) subjects with T2DM had a lower risk of excess mortality compared with those age/sex-matched general subjects in the control group, whereas those with BMI ≥ 40 kg/m^2^ had a substantially higher risk.

The dose–response curve showed that the risk of T2DM decreased as CVAI increased when the latter fell within a lower range, which should be interpreted with caution. Most of the previous studies focused on the relationship between T2DM and obesity instead of low body fat. Adipose tissue plays an important role in energy storage and other biological processes (22), including TG storage and secretion of proteins with multiple functions. Either too little or too much adipose tissue would possibly cause metabolic disorders and changes in insulin sensitivity. Previous studies suggested that patients with adipose tissue deficiency (lipodystrophy) would possibly have limited TG storage in adipose tissue and would redistribute the fat into skeletal muscle and liver (23, 24). Ectopic fat accumulation might contribute to severe insulin resistance, fatty liver, hypertriglyceridemia, and T2DM. Kursawe et al. (25) suggested that the reduction of adipogenesis in adolescents might cause abnormal distribution of abdominal fat, hepatic steatosis, and insulin resistance. Interestingly, Kimet al. (26) found in animal experiments that adipose tissue could be a buffer against risk factors for metabolic disorders. Through appropriate overexpression of adiponectin, adipose tissue would possibly lower the TG level in the liver and muscle and redistribute those TG into subcutaneous adipose tissue, so as to reduce the expression of proinflammatory cytokine and improve insulin sensitivity. The appropriate CVAI level could possibly lower the risk of T2DM and protect the body.

When CVAI fell within a higher range, the cumulative risk of T2DM increased gradually as CVAI increased. It suggested that a higher risk of developing T2DM was associated with higher content of visceral fat when the latter exceeded a specific cutoff point. Too much visceral fat was one of the recognized risk factors for T2DM. Adipose tissue, an important endocrine organ, was a source of multiple proinflammatory cytokines including IL-6 and TNF-α (27). Excessive accumulation of adipose tissue would upregulate the expression of inflammatory factors, resulting in the development of T2DM. Moreover, as hormones expressed in adipose tissue, adiponectin, leptin, and resistin were all closely associated with insulin resistance.

According to the results of the subgroup analysis, there were gender differences in the relationship between CVAI and the risk of T2DM. A higher quartile in CVAI was associated with a higher risk of T2DM for women, but not for men. There were some possible explanations. First, estrogen plays an important role in regulating insulin sensitivity and metabolic homeostasis. It can lower the inflammation levels in white adipose tissue and reduce the levels of immune cell infiltration and oxidative stress in adipose tissue, which reduce possible ectopic lipids in the liver and skeletal muscle (28, 29). Middle-aged and elderly women are losing the protection of estrogen and are more susceptible to insulin resistance than men. Second, Koutsari (30) suggested that women had greater rates of non-oxidative free fatty acid (FFA) disposal than men. The non-oxidative disposal of FFA could possibly increase very low-density lipoprotein (VLDL) and TG and also cause insulin resistance, resulting in metabolic disorders (31–33). Last, previous studies suggested that there are significant differences in the size of adipocytes, basal lipolysis, and fatty acid oxidation rate between women and men. Compared with men, women have lower basal fatty acid oxidation levels and are more susceptible to metabolic disorders (34).

According to our findings, the U-shaped relationship between CVAI and T2DM risk might implicate that it was important to maintain proper visceral fat content. The lowest visceral fat content might not guarantee the best health state. A reduction in visceral fat content was recommended if the baseline CVAI was high, whereas an appropriate increase in visceral fat content might contribute to lowering the risk of T2DM if the baseline CVAI was low. A prospective cohort study (35) suggested that the mortality of diabetic patients decreased as BMI increased, independent of confounders such as smoking, cardiovascular disease, cancer, and other potential factors. Therefore, we suggested that CVAI should be maintained within an appropriate range, which could possibly lower the risk of developing T2DM and reduce the mortality risk associated with T2DM. CVAI at either a very high or a very low level might be detrimental.

### 4.1 Strengths

As a nationwide population-based prospective cohort study, this work covered a wide range of participants with good sample representativeness. We used CVAI for the first time as a continuous variable to explore its dose–response relationship with the risk of T2DM. We built three models to adjust the possible effect of 10 factors (age, gender, education level, location, marital status, smoking status, drinking status, sleep time, SBP, and DBP) on the outcome, which made the study results reliable and convincing. We performed a stratified analysis to explore the differences in the association of CVAI with T2DM risk in multiple subgroups.

### 4.2 Limitations

First, although multiple covariates were adjusted in our study, there were still other potential confounders, such as diet and exercise time. Second, a 2-h oral glucose tolerance test was not performed to detect newly developed T2DM cases, which might underestimate the incidence. Last, we only included participants aged 45 years and above, so further research would be required to generalize our results among younger populations.

## 5 Conclusions

CVAI was closely associated with the risk of T2DM and could possibly be a potential marker in predicting T2DM development. The results might indicate that it would be better to maintain CVAI within an appropriate range.

## Data availability statement

The original contributions presented in the study are included in the article/**Supplementary Material**. Further inquiries can be directed to the corresponding authors.

## Author contributions

GM, SY, LP, and QX: Conception and design of the study, acquisition and interpretation of data, drafting the article. JML: Conception and design of the study, formal analysis and Methodology. YG, JL, HP, LC, and MW: Draw Figures and tables, drafting the article. All authors contributed to and have approved the final manuscript.

## Funding

The work was supported by the Sichuan Provincial Science & Technology Program (2022JDKP0040), Sichuan Provincial Health Commission Program (21PJ168), and Deyang Municipal Science & Technology Program (2021SZZ068, 2022SCZ089 and 2022SCZ130). The funding sources had no role in the study design, data collection, analysis and interpretation of data, decision to publish, or preparation of the manuscript.

## Conflict of interest

The authors declare that the research was conducted in the absence of any commercial or financial relationships that could be construed as a potential conflict of interest.

## Publisher’s note

All claims expressed in this article are solely those of the authors and do not necessarily represent those of their affiliated organizations, or those of the publisher, the editors and the reviewers. Any product that may be evaluated in this article, or claim that may be made by its manufacturer, is not guaranteed or endorsed by the publisher.
